# D6 blastocyst transfer on day 6 in frozen-thawed cycles should be avoided: a retrospective cohort study

**DOI:** 10.1186/s12884-020-03224-z

**Published:** 2020-09-07

**Authors:** Huiling Xu, Shumin Qiu, Xiaojing Chen, Suqin Zhu, Yan Sun, Beihong Zheng

**Affiliations:** Reproductive Medicine Center, Fujian Maternity and Child Health Hospital, Affiliated Hospital of Fujian Medical University, No.18 Daoshan Road, Fujian Province 350001 Fuzhou City, China

**Keywords:** Frozen-thawed cycles, Blastocyst, Timing to transfer blastocyst, Endometrial preparation, Early miscarriage

## Abstract

**Background:**

There is no definitive evidence about the suitable timing to transfer blastocysts formed and cryopreserved on day 6 (D6 blastocysts) in frozen-thawed embryo transfer (FET) cycles. This study aimed to investigate the suitable timing to transfer D6 blastocysts in FET cycles and to identify factors affecting clinical pregnancy rate (CPR) and early miscarriage rate (EMR) in FET cycles with blastocysts.

**Methods:**

This retrospective cohort study included 1788 FET cycles with blastocysts. There were 518 cycles with D6 blastocysts, and 1270 cycles with blastocysts formed and cryopreserved on day 5 (D5 blastocysts) (D5 group). According to the blastocyst transfer timing, the cycles with D6 blastocysts were divided into cycles with D6 blastocysts transferred on day 5 (D6-on-D5 group, 103 cycles) and cycles with D6 blastocysts transferred on day 6 (D6-on-D6 group, 415 cycles). The chi-square test, independent t-test or Mann-Whitney test, and logistic regression analysis were used for data analysis.

**Results:**

The CPR and implantation rate (IR) were significantly higher in the D6-on-D5 group than in the D6-on-D6 group (55.3% vs. 37.3%, 44.8% vs. 32.6%, *P* < 0.01). The CPR and IR were significantly higher in the D5 group than in the D6-on-D5 group (66.0% vs. 55.3%, 62.1% vs. 44.8%, *P* < 0.05), and the EMR was significantly lower in the D5 group than in the D6-on-D5 group (11.2% vs. 21.1%, *P* < 0.05). Logistic regression analysis demonstrated that transfer D6 blastocysts on day 5, instead of day 6, could significantly increase the CPR (odds ratio[OR]: 2.031, 95% confidence interval (CI): 1.296–3.182, *P* = 0.002). FET cycles with D6 blastocysts transferred on day 5 had a higher EMR than those with D5 blastocysts (OR: 2.165, 95% CI: 1.040–4.506, *P* = 0.039). Hormone replacement therapy (HRT) cycles exhibited a higher EMR than natural cycles (OR: 1.953, 95% CI: 1.254–3.043, *P* = 0.003), while no difference was observed in the CPR (*P* > 0.05).

**Conclusions:**

These results indicate that the suitable timing to transfer D6 blastocysts in FET cycles may be day 5, and D6 blastocyst transfer on day 6 in FET cycles should be avoided. D6 blastocysts transfer and HRT cycles may be associated with a higher EMR.

## Background

Blastocyst transfer facilitates a high pregnancy rate and reduces the incidence of multiple pregnancy and ectopic pregnancy[1–6]. Prolonged culture of the embryos to blastocyst stage has become the preferred method in most reproductive centers[5, 6]. Prospective randomized controlled trials have reported a higher implantation rate(IR) and clinical pregnancy rate(CPR) in frozen-thawed embryo transfer (FET) cycles [7, 8]. A better embryo-endometrium synchronization is observed in FET cycles rather than fresh cycles[7–9]. At the same time, cryopreservation has become an extraordinarily important step to minimize the risk of ovarian hyper-stimulation syndrome and maximize the efficacy of ovarian stimulation cycles in the assisted reproductive technology (ART). Over the past several decades, vitrification/warming has been proven to be superior to low-freezing/thawing with regard to cryosurvival rates and clinical outcomes, and it has become the favoured option for embryo cryopreservation[10]. As a result, the use of vitrified blastocyst transfer is becoming increasingly widespread.

Many studies have examined the outcomes of FET cycles with blastocysts. Some reports showed equivalent outcomes between blastocysts formed and cryopreserved on day 5 (D5 blastocysts) and blastocysts formed and cryopreserved on day 6 (D6 blastocysts)[11–14]. Meanwhile, there is a growing body of evidence indicating that the CPR, IR, and live birth rate (LBR) with D5 blastocysts are higher than with D6 blastocysts in FET cycles[15–20]. Therefore, whether the day of blastocyst formation affects clinical outcomes remains controversial. The factors affecting CPR have not been clarified yet, and few studies have focused on the early miscarriage rate(EMR).

For successful implantations, receptive endometrium and synchronization between the endometrium and embryos are essential. Several studies have provided evidence that in fresh cycles blastocysts transfer on day 6 should be avoided, and it should be performed on day 5[9, 14, 21]. However, there is no definitive evidence about the suitable timing to transfer D6 blastocysts in FET cycles. In most reproductive centers, D5 blastocysts are believed to be developing at a normal rate, whereas D6 blastocysts are believed to be developing slowly. Therefore, both D5 and D6 blastocysts are scheduled to be transferred on 5 days after ovulation or progesterone use in hormone replacement therapy (HRT) cycles (day 5 in FET cycles)[11, 15, 22]. Occasionally, D6 blastocysts may be transferred on 6 days after ovulation in natural FET cycles to avoid transfers on the weekends. However, in other reproductive centers, including our center, it is believed that the D3 embryos should be transferred on day 3, D5 blastocysts should be transferred on day 5, and therefore D6 blastocysts should be synchronized with the endometrium on day 6 logically and they are planned to be transferred on day 6. Both hypotheses about the timing to transfer D6 blastocysts sound reasonable.

This study aimed to investigate the suitable timing to transfer D6 blastocysts in FET cycles, and identify the factors affecting CPR and EMR in FET cycles with blastocysts.

## Methods

### Ethics statements

This study was approved by the Ethics Committee of Fujian Maternity and Child Health Hospital (approval number: 2019 − 2008).

### Study design and patients

This retrospective cohort study included 1788 FET cycles with blastocysts formed and vitrified on either day 5 or day 6 and transferred between June 2017 and November 2018. There were 518 cycles with D6 blastocysts and 1270 cycles with D5 blastocysts (D5 group). According to the blastocyst transfer timing, the cycles with D6 blastocysts were divided into cycles with D6 blastocysts transferred on day 5 (D6-on-D5 group, 103 cycles) and cycles with D6 blastocysts transferred on day 6 (D6-on-D6 group, 415 cycles).

### Fertilization methods, embryo culture and scoring

Conventional in vitro fertilization (IVF) or intracytoplasmic sperm injection was performed as appropriate based on the absence or presence of male factor infertility. Embryos were cultured individually in 50 µl of culture medium droplets (Vitrolife, Sweden) under mineral oil (SAGE, Denmark) and incubated in an atmosphere of 6.0% carbon dioxide, 5% oxygen and 89% nitrogen. Fertilization was assessed at 16–18 hours after post insemination/injection and an embryo quality assessment was performed on day 2 and day 3. After one to two embryos were transferred or vitrified on day 3, all of the supernumerary embryos were transferred to G-2™ PLUS medium (Vitrolife, Sweden) for extended culture under the same conditions until day 5 or day 6, regardless of their quality.

Blastocyst quality was assessed on day 5 and day 6 according to the Gardner scoring system, taking into account the degree of expansion, the quality of the inner cell mass, and the quality of the trophectoderm cells[23]. Blastocysts with a Gardner score of 3BB or better were considered to be good quality, and other blastocysts with a Gardner score better than 3CC were considered to be qualified; all of these blastocysts were considered to be suitable for cryopreservation by vitrification. Blastocysts with a Gardner score better than 3CC on day 5 were vitrified, and the other embryos were cultured until day 6. On day 6, blastocysts with a Gardner score better than 3CC were vitrified and the rest were discarded.

### Blastocyst vitrification and thawing procedures

Blastocysts vitrification was performed using vitrification straws ( JY Straws ,Canada) in combination with a vitrification kit(KITAZATO, Japan). Then each straw containing one or two blastocysts was plunged into liquid nitrogen.

The objective of our laboratory protocol was to transfer D5 blastocysts first. Thereafter, the choice of the blastocyst to be transferred was based on its quality according to the Gardner scoring system. On the day of the embryo transfer, the blastocyst was thawed using the thaw kit (KITAZATO, Japan). After warming, the blastocyst quality was evaluated when re-expansion occurred. If inner cell mass(ICM) and/or trophectoderm lysis was present, the blastocyst was discarded and another one was warmed. After survival assessment, the blastocyst was cultured in G-2™ PLUS medium (Vitrolife, Sweden) for 1–2 hours before transfer under ultrasound guidance.

### Endometrial preparation and thawed embryo transfer

FET was performed with a natural cycle for patients with regular ovulation. Ovulation was determined as follows. Follicular development was monitored by vaginal ultrasonography from the 10th to 12th day of the menstrual cycle, and once the follicle reached a diameter of 14 mm, the urinary luteinizing hormone(LH) level was monitored. If the follicle reached a diameter of 16–18 mm with blood appearance of an LH level peak, vaginal ultrasonography was performed at 24–48 hours after the LH peak, the absence of the follicle was defined as ovulation. Patients received an oral administration of 10 mg of dydrogesterone (Duphaston, Abbott, Netherlands) twice daily after ovulation until the β-subunit of human chorionic gonadotropin (β-hCG) test was performed. D5 blastocysts were transferred 5 days after ovulation, and D6 blastocysts were transferred 5 or 6 days after ovulation. If ovulation did not occur owing to luteinized unruptured follicle syndrome, we transferred D5 blastocysts 6 days after the LH peak, and D6 blastocysts 6 or 7 days after the LH peak.

For patients without regular ovulation or whose endometrium thickness was ≤ 8 mm after ovulation, FET was conducted with an HRT cycle. The patients initially received an oral administration of 4–12 mg/day of estradiol (Progynova, Bayer, Germany) for endometrial preparation from the 2nd to 5th day of the cycle. The starting dose was set according to the patient’s previous endometrial conditions. An ultrasonographic endometrial assessment was performed approximately 10 days later. For patients with an endometrial thickness of more than 8 mm, natural vaginal progesterone (Crinone vaginal gel, Merck Serono, UK) at a dose of 90 mg per day in the morning and 10 mg of dydrogesterone (Duphaston, Abbott, Netherlands) twice daily weradded. If the endometrial thickness was not adequate, the dose of estradiol was increased and an ultrasonographic assessment was conducted to confirm further endometrial thickening. D5 blastocysts were transferred 5 days after progesterone use, and D6 blastocysts were transferred 5 or 6 days after progesterone use. After the transfer, the use of oestrogen and progesterone was continued for 14 days until the β-hCG test was performed.

There were 10 physicians in our center; 8 believed that D6 blastocysts transfer on day 6 would be better for the patients, and 2 believed that D6 blastocysts should be transferred on day 5. The physicians alternated shifts. The timing to transfer D6 blastocysts was determined by the belief of the physician on duty for deciding the transfer time, regardless of blastocyst quality or any other factors.

Embryo transfer was performed under ultrasound guidance; one or two blastocysts were transplanted per cycle.

### Determination of pregnancy

The serum β-hCG level was measured 14 days after embryo transfer. A biochemical pregnancy was defined as a β-hCG level > 25 U/L. In case of pregnancy, progesterone treatment with or without oestrogen was maintained at the same dose until the first vaginal ultrasonography was performed 28–35 days after transfer to confirm the clinical pregnancy. Subsequently, the administration of vaginal natural progesterone (Crinone vaginal gel, Merck Serono, UK) was discontinued, the dose of oestrogen was tapered, and administration of dydrogesterone (Duphaston, Abbott, Netherlands) was continued for the women who became pregnant until the 10th to 12th week of pregnancy.

### Outcome measures

The main outcome measures were the CPR, IR and EMR. Clinical pregnancy was defined as visualization of a gestational sac. The IR was defined as the ratio between the number of gestational sacs observed under B ultrasonography and the number of blastocysts transferred. Early miscarriage was defined as an intrauterine pregnancy loss before 13 weeks of gestation. Live birth data were not available for ongoing pregnancies at the time of analysis.

### Statistical analysis

We used the following tests: the chi-square test to analyse categorical variables, the independent t-test or Mann-Whitney test to analyse continuous variables, as appropriate, and logistic regression analysis for the multivariate analysis. We considered maternal age, paternal age, body mass index (BMI), type of infertility, previous failures, endometrial thickness, endometrial preparation, physician performing the transfers, number of blastocysts transferred, quality of blastocysts transferred and category of blastocyst transfer as potential confounders. Among these, the type of infertility, previous failures, endometrial preparation, physician performing the transfers, number of blastocysts transferred, quality of blastocysts transferred and category of blastocyst transfer were considered as categorical data. Categories of blastocyst transfer included D6 blastocysts transferred on day 6 (D6-on-D6), D6 blastocysts transferred on day 5 (D6-on-D5) and D5 blastocysts transferred on day 5(D5). We constructed the multivariable logistic regression model using the enter method. All statistical analyses were performed using the software package SPSS 19.0(IBM Corp., Armonk, NY, USA). *P* < 0.05 was considered to be statistically significant.

## Results

In total, 1788 FET cycles with blastocysts were performed, and 2185 vitrified blastocysts were thawed, including 2166 blastocysts that survived and were transferred (with blastocoelic expansion); the survival rate was 99.1%. Similar survival rates were observed for D5 and D6 blastocysts (99.0% [1471/1486] and 99.4% [695/699], respectively, P > 0.05). Overall, 47.4% (848/1788) of the couples had primary infertility, and the others had secondary infertility. The average maternal age was 31.54 ± 4.49 years (range: 20–45 years), and the average number of blastocysts transferred was 1.21 ± 0.41.

We compared patient characteristics between the D6-on-D5 group and D6-on-D6 group (Table 1). There were no significant differences in maternal age, paternal age, BMI, proportion of primary infertility, proportion of natural cycles for endometrial preparation, proportion of good quality blastocysts or proportion of single blastocyst transfer (SBT) between the groups. The endometrial thickness after ovulation in natural cycles or before the administration of progesterone in HRT cycles was comparable between the groups. The average number of blastocysts transferred per cycle was also similar between the groups. The CPR and IR were significantly higher in the D6-on-D5 group than in the D6-on-D6 group (55.3%vs. 37.3%, 44.8% vs. 32.6%, *P* < 0.01) (Table 1).

**Table 1:** Comparison of FET cycles between the D6-on-D5 group and D6-on-D6 group

	D6-on-D5 group(*n* = 103)	D6-on-D6 group(*n* = 415)	*P*-value
Maternal age(years)	31.89 ± 4.46	31.96 ± 4.68	0.890
Paternal age (years)	33.88 ± 4.97	34.08 ± 5.25	0.726
BMI(kg/m^2^)	21.23 ± 2.67	21.27 ± 2.70	0.896
Endometrial thickness (mm)	9.82 ± 1.37	9.77 ± 1.40	0.746
Number of blastocysts transferred	1.39 ± 0.49	1.33 ± 0.47	0.278
Type of infertility			
Primary infertility	41.7% (43)	48.4%(201)	0.224
Secondary infertility	58.3% (60)	51.6%(214)	
Endometrial preparation			
Natural cycles	38.8% (40)	38.8% (161)	0.994
HRT cycles	61.2% (63)	61.2% (254)	
Number of blastocyst transferred			
Single blastocyst transfer	61.2% (63)	67.0% (278)	0.265
Double blastocyst transfer	38.8% (40)	33.0% (137)	
Quality of blastocyst transferred			
Qualified	41.7% (43)	45.1% (187)	0.545
Good	58.3% (60)	54.9% (228)	
Clinical pregnancy rate (%)	55.3(57/103)	37.3(155/415)	0.001
Implantation rate (%)	44.8(64/143)	32.6(180/552)	0.007
Early miscarriage rate (%)	21.1(12/57)	16.7(26/155)	0.471
Multiple pregnancy rate (%)	12.3(7/57)	16.1(25/155)	0.488

Data are presented as mean ± standard deviation (SD) or % (n )

*BMI* body mass index, *HRT* hormone replacement therapy, *FET* frozen-thawed embryo transfer, *D6-on-D5 group* cycles with blastocysts formed and cryopreserved on day 6 that were transferred on day 5, *D6-on-D6 group* cycles with blastocysts formed and cryopreserved on day 6 that were transferred on day 6

When comparing the D5 group and D6-on-D5 group, we found that maternal age, paternal age, BMI, endometrial thickness, and proportions of primary infertility patients and natural cycles were comparable between the groups. The proportion of SBT and the proportion of good quality blastocysts were significantly higher in the D5 group than in the D6-on-D5 group (84.2% vs. 61.2%, 87.6% vs.58.3%, *P* = 0.000); thus, more blastocysts were transferred in the D6-on-D5 group than in the D5 group per cycle (1.39 ± 0.49 vs. 1.16 ± 0.37, *P* = 0.000). However, the CPR and IR were significantly higher in the D5 group than in the D6-on-D5 group (66.0% vs. 55.3%, *P* = 0.029, 62.1% vs. 44.8%, *P* = 0.000), and the EMR was significantly lower in the D5 group than in the D6-on-D5 group (11.2% vs. 21.1%, *P* = 0.026) (Table 2)

**Table 2:** Comparison of FET cycles between the D5 group and D6-on-D5 group

	D5 group(*n* = 1270)	D6-on-D5 group(*n* = 103)	*P*-value
Maternal age(years)	31.37 ± 4.42	31.89 ± 4.46	0.254
Paternal age (years)	33.54 ± 5.08	33.88 ± 4.97	0.507
BMI(kg/m^2^)	21.22 ± 2.74	21.23 ± 2.67	0.978
Endometrial thickness(mm)	9.72 ± 1.46	9.82 ± 1.37	0.466
Number of blastocysts transferred	1.16 ± 0.37	1.39 ± 0.49	0.000
Type of infertility			
Primary infertility	47.6%(604)	41.7% (43)	0.256
Secondary infertility	52.4%(666)	58.3% (60)	
Endometrial preparation			
Natural cycles	40.6% (515)	38.8% (40)	0.733
HRT cycles	59.4% (755)	61.2% (63)	
Number of blastocyst transferred			
Single blastocyst transfer	84.2% (1069)	61.2% (63)	0.000
Double blastocyst transfer	15.8% (201)	38.8% (40)	
Quality of blastocysts transferred			
Qualified	12.4% (157)	41.7% (43)	0.000
Good	87.6% (1113)	58.3% (60)	
Clinical pregnancy rate (%)	66.0(838/1270)	55.3(57/103)	0.029
Implantation rate (%)	62.1(913/1471)	44.8(64/143)	0.000
Early miscarriage rate (%)	11.2(94/838)	21.1(12/57)	0.026
Multiple pregnancy rate (%)	8.9(75/838)	12.3(7/57)	0.399

Data are presented as mean ± SD or % (n)

Multivariate logistic regression analysis was performed to investigate the factors affecting CPR and EMR. The variables entered into the logistic regression model included maternal age, paternal age, BMI, type of infertility, previous failures, endometrial thickness, endometrial preparation, physician performing the transfers,number of blastocysts transferred, quality of blastocysts transferred and category of blastocyst transfer. The analysis demonstrated that transfer D6 blastocysts on day 5, instead of day 6, significantly increased the CPR (odds ratio [OR]: 2.031, 95% confidence interval (CI): 1.296–3.182, *P* = 0.002). Maternal age was negatively associated with the CPR (OR:0.957, 95% CI: 0.922–0.993, *P* = 0.020). Double blastocyst transfer increased the CPR (OR:1.502, 95% CI: 1.164–1.939, *P* = 0.002), similar to the transfer of good quality blastocysts (OR:1.631, 95% CI: 1.266–2.101, *P* = 0.000). FET cycles with D6 blastocysts transfer on day 5 exhibited a higher EMR than FET cycles with D5 blastocysts (OR: 2.165, 95% CI: 1.040–4.506, *P* = 0.039). HRT cycles exhibited a higher EMR than natural cycles (OR: 1.953, 95% CI: 1.254–3.043, *P* = 0.003), while no significant difference was observed in the CPR(*P* > 0.05). (Tables 3 and 4).

**Table 3:** Multivariable logistic regression analysis of the factors affecting CPR

	OR	95% CI	*P*-value
Maternal age	0.957	0.922–0.993	0.020
Paternal age	1.006	0.974–1.039	0.712
BMI	1.020	0.983–1.058	0.295
Type of infertility	1.015	0.825–1.250	0.885
Previous failures^a^			0.202
Endometrial thickness	1.074	0.995–1.160	0.066
Endometrial preparation	0.995	0.797–1.242	0.966
Physician performing the transfers^a^			0.969
Number of blastocyst transferred	1.502	1.164–1.939	0.002
Quality of blastocysts transferred	1.631	1.266–2.101	0.000
Category of blastocyst transfer			0.000
D5 versus D6-on-D6	2.951	2.289–3.805	0.000
D6-on-D5 versus D6-on-D6	2.031	1.296–3.182	0.002

*OR* odds ratio, *CI* confidence interval, *BMI* body mass index

The total sample size of the logistic model was 1788 FET cycles with blastocysts

^a^The number of dummy variables is excessively long to list

**Table 4:** Multivariable logistic regression analysis of the factors affecting EMR

	OR	95% CI	*P*-value
Maternal age	1.032	0.965–1.105	0.357
Paternal age	1.035	0.978–1.095	0.233
BMI	1.025	0.957–1.098	0.478
Type of infertility	0.999	0.674–1.480	0.995
Previous failures^a^			0.996
Endometrial thickness	0.988	0.851–1.146	0.869
Endometrial preparation	1.953	1.254–3.043	0.003
Physician performing the transfers^a^			0.991
Number of blastocyst transferred	1.633	0.981–2.719	0.059
Quality of blastocysts transferred	1.368	0.841–2.226	0.207
Category of blastocyst transfer			0.042
D6-on-D6 versus D5	1.636	0.971–2.757	0.065
D6-on-D5 versus D5	2.165	1.040–4.506	0.039

The total sample size of the logistic model was 1050 clinical pregnant FET cycles

^a^The number of dummy variables is excessively long to list

## Discussion

Shapiro et al. observed that the ongoing pregnancy rate for D6 blastocyst transfer in fresh cycles was significantly lower than that in FET cycles (17.1% vs. 54.3%) [14]. After ovarian stimulation, the implantation window was advanced, while D6 blastocyst was slower-growing, both of these factors lead to a worse embryo-endometrium synchronization[9]. Moreover, in order to exclude the possible impairment of embryo quality with slow developing blastocysts, Poulsen et al. demonstrated that even elective blastocyst transfer on day 6 was associated with a lower IR than day 5 transfer in fresh cycles(29.9% vs. 55.1%)[21]. These studies indicated that blastocyst transfers on day 6 in fresh cycles should be avoided because of decreased endometrial receptivity. To date, most D6 blastocysts are transferred in FET cycles.

However, the important question of the suitable timing to transfer D6 blastocysts in FET cycles has not been well studied. Our analysis is the first large research to focus on the effects of different timing to transfer D6 blastocysts in FET cycles on clinical outcomes. We found that when D6 blastocysts were transferred on day 5, instead of day 6, the IR and CPR were significantly higher. Because the characteristics of the patients and blastocysts were comparable between the groups, the difference in IR and CPR can be assumed to reflect the difference in endometrial receptivity only and be unaffected by other factors. In other words, endometrial receptivity for D6 blastocysts is higher on day 5 than on day 6. The delayed blastocyst formation of D6 blastocysts results in better synchrony with endometrial development on day 5. Our results indicate that day 5 is the suitable timing to transfer D6 blastocysts in FET cycles, and D6 blastocysts transfer on day 6 should be avoided.

Whether the day of blastocyst formation affects clinical outcomes is still controversial. Some reports have shown that D6 blastocysts have no effect on the CPR or LBR [11, 12]. In Yang et al.’s study, when only high-quality blastocysts were transferred, the CRP and IR of D5 and D6 blastocysts were similar[18]. However, when all the blastocysts were taken in to account, the CRP and IR of the D5 blastocysts were higher than those of the D6 blastocysts [18]. Hass et al. demonstrated that even the CPR of morphologically good quality D6 blastocysts is significantly lower than that of D5 blastocysts in FET cycles[16]. Additionally, Ferreux et al. reported that no matter what the blastocyst quality was, the LBR of D6 blastocysts following FET was significantly lower than that of D5 blastocysts [15]. Our results are consistent with these findings regarding the higher CPR and IR with D5 blastocysts than with D6 blastocysts in FET cycles. However, the proportion of good quality blastocysts was much higher in the D5 group than in D6-on-D5 group.

Moreover, we demonstrated that D6 blastocysts exhibited a higher EMR than D5 blastocysts. A meta-analysis showed no significant difference in EMR between D5 and D6 blastocyst transfers in FET cycles[12]. Ferreux et al. also reported similar EMRs between D5 and D6 cryopreserved blastocyst transfers[15]. However, similar to our study, Wang et al. reported a higher EMR in the D6 blastocysts than in the D5 blastocysts(16.4% vs. 11.9%), although not statistically significant[19].

Chromosomal abnormalities are highly correlated with implantation failure and early miscarriages[24]. Campbell et al. reported that aneuploid blastocysts had delayed initiation of blastulation compared with euploid blastocysts by using a time-lapse culture system [25]. Alfarawati et al. found that slower developing blastocysts had higher rates of aneuploidy[26]. Zhan et al. reported a significant higher euploid rates in D5 blastocysts than in D6 blastocysts (51.6% vs. 34.0%)[27]. Taylor et al. also found that euploid rates were significantly higher in D5 blastocysts than in D6 blastocysts (55.8% vs. 44.6%,*P =* 0.0014), and when only euploid D5 or D6 blastocysts were transferred during FET cycles, there were no significant differences between them regarding the CPR, IR, or ongoing/live birth rates[28]. Similarly, Capalbo et al. found that the ongoing pregnancy rate of euploid D5 and D6 blastocysts was similar(48.8% and 51.2%, respectively)[29]. In the present study, preimplantation genetic screening was not performed; therefore, there may have been more aneuploidy blastocysts in the D6-on-D5 group than in the D5 group. A difference in aneuploidy rates between D5 and D6 blastocysts would explain why D5 blastocysts transfer in our study were associated with better clinical outcomes (i.e.--- higher CPR and IR, and lower EMR).

There are also some alternative explanations for the lower IR of D6 blastocysts. For instance, Hashimoto et al. reported a higher incidence of abnormal spindles and a lower IR in D6 blastocysts than in D5 blastocysts[30]. However, although giving priority to D5 blastocysts would shorten the time to pregnancy, the transfer of cryopreserved D6 blastocysts remains a viable and important option.

Many studies have found no significant differences in the CPR, IR, or LBR between the HRT and natural cycles in FET[31–33]. However, we found that although no significant difference was observed in the CPR of HRT cycles or natural cycles, HRT cycles were associated with a higher EMR, which is consistent with the findings reported by Maria et al. In their prospective study, they observed a higher miscarriage rate in the HRT group[34].

There are limitations of the present study that should be acknowledged. First, we did not use an SBT strategy. Furthermore, we did not divide the patients undergoing D6 blastocysts transfer into different groups randomly. We divided patients based on the involved physicians’ opinions, which resulted in an unequal number of cycles in the two groups; this may be a significant confounding variable. Additionally, although this study included nearly 1 800 blastocyst transfers, it was nevertheless a retrospective analysis. However, the significant reduction in the CPR observed in the study makes a prospective randomized study between day 5 and day 6 transfer of D6 blastocysts ethically difficult.

## Conclusions

In conclusion, the present study’s findings suggest that the suitable timing to transfer D6 blastocysts in FET cycles may be day 5. Thus, D6 blastocysts transfer on day 6 should be avoided. D6 blastocysts transfer and HRT cycles may be associated with a higher EMR. Further researches are needed to elucidate the mechanism.

## Data Availability

The datasets used and analysed during the current study are available from the corresponding author on reasonable request.

## Abbreviations

CPR: Clinical pregnancy rate
IR: Implantation rate
FET: Frozen-thawed embryo transfer
D5 blastocysts: Blastocysts formed and cryopreserved on day 5
D6 blastocysts: Blastocysts formed and cryopreserved on day 6
LBR: Live birth rate
EMR: Early miscarriage rate
HRT: Hormone replacement therapy
IVF: In vitro fertilization
D5 group: Cycles that included blastocysts formed and cryopreserved on day 5
D6-on-D5 group: Cycles with blastocysts formed and cryopreserved on day 6 that were transferred on day 5
D6-on-D6 group: Cycles with blastocysts formed and cryopreserved on day 6 that were transferred on day 6
LH: Luteinizing hormone
β-hCG: β-subunit of human chorionic gonadotropin
BMI: Body mass index
SBT: Single blastocyst transfer
OR: Odds ratio
CI: Confidence interval
